# Training doctors briefly and in situ to involve their patients in making medical decisions—Preliminary testing of a newly developed module

**DOI:** 10.1111/hex.12565

**Published:** 2017-05-18

**Authors:** Jürgen Kasper, Katrin Liethmann, Christoph Heesen, Daniel R Reissmann, Friedemann Geiger

**Affiliations:** ^1^ Department Health and Caring Sciences Faculty of Health Sciences The Arctic University of Norway Tromsø Norway; ^2^ Medical Clinics University Medical Center Tromsø Norway; ^3^ Unit of Health Sciences and Education Faculty of Mathematics Informatics and Natural Sciences University of Hamburg Hamburg Germany; ^4^ Department of Neurology Institute of Neuroimmunology and Multiple Sclerosis University Medical Center Hamburg‐Eppendorf Hamburg Germany; ^5^ Institute of Dental Prosthetics University Medical Center Hamburg‐Eppendorf Hamburg Germany; ^6^ MSH Medical School Hamburg Hamburg Germany; ^7^ Department of Pediatrics University Medical Center Schleswig‐Holstein Kiel Germany; ^8^ Institute of Medical Psychology and Sociology University Medical Center Schleswig‐Holstein Kiel Germany

**Keywords:** doctor‐patient relation, evidence‐based medicine, medical education, shared decision making, training

## Abstract

**Objective:**

To carry out preliminary evaluation of a training module for doctors to enhance their ability to involve their patients in medical decision making. The training refers to the shared decision‐making (SDM) communication concept.

**Methods:**

The training module includes a comprehensive manual, a corresponding video tutorial with communication examples and a 15‐minute face‐to‐face feedback session based on an SDM analysis of a consultation recording provided by the trainee. Ten trainees (four neurologists, three dentists, and three general practitioners) participating in the pretest each recorded four clinical consultations (total sample: N=40) and received three training components. After the training, doctors provided feedback on the module's feasibility in a questionnaire. Communication performance of doctors, patients and doctor–patient dyads was assessed by trained observers and self‐assessed by doctors and patients using the MAPPIN’SDM approach. Training effects were determined using Wilcoxon signed‐rank tests comparing baseline values with post‐intervention performance as assessed in the fourth consultations.

**Results:**

The face‐to‐face training sessions were short and feasible with regard to clinical reality. Participants considered the training supportive for acquiring SDM skills and recommended more emphasis on the face‐to‐face feedback. Communication improved according to observers rating doctors (*P*=.05) and doctor–patient dyads (*P*=.07) and to doctors’ own judgements (*P*=.02). No improvement was observed in patients’ SDM behaviour (*P*=.11); accordingly, patients’ judgements did not indicate improvement (*P*=.14).

**Conclusions:**

The training is designed to meet clinicians’ needs. Improvement of risk communication after training encourages optimization according to doctors’ feedback. Following this study, the efficacy of the training is now being examined in a randomized controlled trial.

## 1 BACKGROUND

Three decades of research on shared decision making have not been enough to provide unambiguous proof that shared decision making (SDM) leads to patient‐relevant outcomes,^1^, ^2^ such as better health outcomes, lower decisional conflict, and better adherence. According to a recent review, in 10 (out of 11) randomized trials which succeeded in increasing patient participation, only six (14%) of the 42 hypothesized outcomes were found to be positively influenced.^1^ Perhaps, we should prepare ourselves to accept that shared decision making is justified by an ethical imperative only rather than empirical evidence.^3^, ^4^ Wouldn't this give enough reason to strive for its implementation? The absence of proof is, however, not proof of absence. Difficulties in demonstrating a benefit for patients from being involved in making their medical decisions could also be due to methodological deficiencies. If effects fail to appear after provision of interventions supposed to facilitate SDM communication, methodological deficiencies might arise in conjunction with insufficient quality of the interventions. Evidence shows neither SDM tutorials^5^ nor patient decision aids^6^ to reliably achieve the intended communication quality. If effects fail to appear although patient participation has demonstrably been achieved, methodological deficiencies might arise from existing measures’ inability to really capture the concept's essentials.^7^, ^8^, ^9^ Given the weakness of existing knowledge on the efficacy of SDM despite the voluminous literature of the last decades, consideration of such methodological deficiencies implies the potential to nevertheless discover the impact of the concept by use of newly developed or refined appropriate methods.

This study was carried out in response to both the communication deficits still present in health care^10^ and the underdeveloped level of SDM training for health professionals.^5^ A systematic review identified 54 training programmes in 14 countries and 10 languages. Only 17 of them had been evaluated, in most cases using trainee satisfaction or other subjectively reported outcomes. The authors concluded that more knowledge is needed on training didactics and on the training programmes’ efficacy with regard to patient‐relevant outcomes.^5^ In addition, the vast majority of the training programmes listed in this review addressed decision making in specific medical domains. Broad‐scale implementation of shared decision making, however, requires generic training methods that can be used in any medical context. When we started developing our training module, no German‐language SDM training for health professionals was available that complied with minimal criteria of feasibility, efficacy and generalizability.

To enhance physicians’ ability to involve patients in the process of making medical decisions, we developed *doktormitSDM*, a short in situ training module. The new module was intended to comply with at least the following three requirements. (i) The training approach should entirely meet the essentials of the SDM concept.^11^, ^12^ To give examples, this includes encouraging professionals to develop willingness to rigorously share all relevant information with the patient and the corresponding competency to adhere to the criteria of evidence‐based patient information.^13^ (ii) To assure feasibility, the training should also comply with health professionals’ practical needs and restrictions, particularly with regard to time resources. (iii) To be adaptable to varying contexts, the didactic concept of the new training module should also allow for a generic approach.

When we conducted this study, doktormitSDM had already undergone some unsystematic piloting and pretesting. Single components were evaluated either using in‐depth interviews with physicians at our own unit or by administering a feedback questionnaire to participants in several conference workshops.^14^, ^15^, ^16^ Particular attention was paid to practical issues, acceptance and subjective perception of usability. An initial rough draft was adapted, incorporating theoretical input, to produce an intervention module consisting of a 15‐minute feedback session, a 20‐minute video tutorial and an SDM manual, which we then considered ready for systematic pretesting.

This study aimed to explore the feasibility of the new training module. In particular, we investigated practicability in the context of the doctors’ clinical practice and whether the doctors considered the course helpful. In addition, the study was intended to help in evaluating the appropriate training dose. Would the use of a minimal intervention be already enough enable doctors to change their communication behaviour? As, in our experience, health professionals’ concerns about changing communication habits are often related to the length of consultations, this study also set out to explore the relationship between communication quality and the use of time.

## 2 METHODS

### 2.1 Design

**Figure 1:**
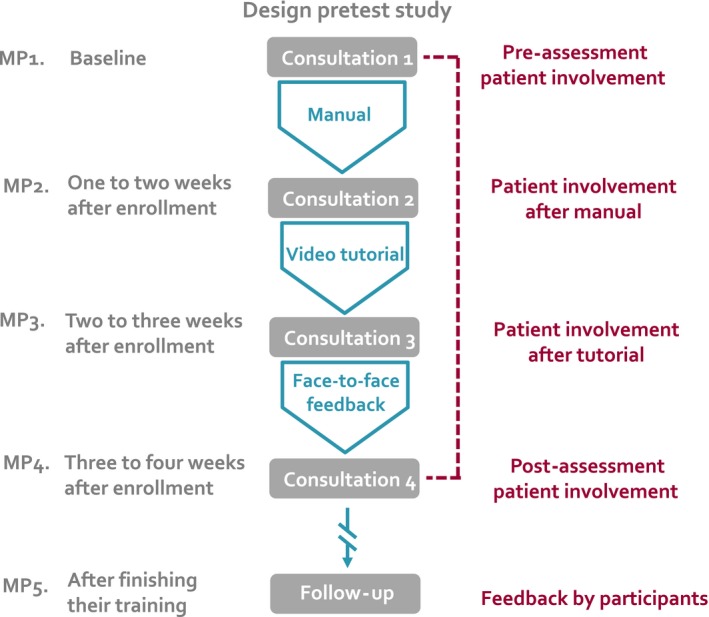
Pretest study design. MP, measurement point

The study used a one‐cohort pre‐post design consisting of an alternating sequence of decisional consultations and intervention components provided stepwise (Figure 1). After the training, feedback on feasibility and perceived benefit was obtained from the participating doctors using a questionnaire. Communication quality was evaluated on the basis of four consultation recordings delivered by each participant. The first consultation was used for baseline assessment. After each of the first three consultations, a component of the intervention was provided. A fourth consultation (C4) was recorded to assess the training effect defined as improvement from baseline to the end of the training. To allow for exploratory elucidation of the learning curve, additional measurement points after the first two intervention components (C2 and C3) were included in the study design. Extent of patient involvement was evaluated from the third person observer's, the doctor's and the patient's perspective. Patients participating in this study were blind towards the doctors’ training level and were only recorded once within the study. For organizational reasons, the doctors were free to choose the consultations for the study. However, the selection was made before the consultations. The pauses between the consultations were supposed to be just long enough to receive the next intervention step, but as brief as possible.

This study was approved by the ethics committee of the Christian‐Albrechts University Kiel, Germany, and was registered in the Current Controlled Trials register (ISRCTN78716079). All participating physicians and patients gave their written consent to inclusion in the study.

### 2.2 The intervention

The doktormitSDM training module is minimized with regard to volume but comprehensive with regard to the didactic approach. This section describes the structure and content of the intervention used in this study. As we also wish to share our knowledge, which has grown over years, the purpose of the following section is threefold: to give a precise scientific description, to provide some narrative background about the genesis and to give insight into didactic considerations related to the SDM training approach evaluated in this study.

#### 2.2.1 Description of the training module

The doktormitSDM training module is included in the environmental review of health professional training in SDM by Légaré et al.^17^ It has three parts:

1. Training manual: Demonstrating transparency, doctors are provided with the manual, used by observers applying the MAPPIN'SDM coding (Multifocal Approach to the ‘Sharing’ in SDM).^18^ It includes comprehensive background on the idea and state of research on SDM. Moreover, as a well‐structured reference framework, the manual illustrates each of a set of 15 SDM skills using examples for different levels of performance.
2. Video tutorial: The tutorial lasts 20 minutes and presents a composition of sequences of real decision consultations from a broad variety of medical domains. The sequences are structured and edited according to the MAPPIN'SDM taxonomy. All examples show good to excellent performance.
3. Face‐to‐face feedback: The 15‐minute feedback session is based on a MAPPIN'SDM analysis of the consultation video provided by the participant. The feedback follows a generic structure but is applied in a highly individual and interactive way. Comments provided within the feedback session never refer to the general communication performance and do not relate to work samples other than the one given in this session.

Participants were invited to use the manual and the tutorial as preparation for their individual training. They were told that these sources provide background about the SDM approach and the particular system used, to structure the communication.^18^ The manual explicitly states that it is written for both raters and clinicians and provides guidance indicating passages that are easy to read and others that are relevant to researchers only. In total, the manual has 40 pages; reading the entire text would have required approximately 2 hours.

#### 2.2.2 Origin and core operation

The training concept uses knowledge gained during the development of MAPPIN'SDM, a measurement inventory to assess SDM.^7^, ^18^, ^19^, ^20^ MAPPIN'SDM had resulted from thorough consideration of the concept's assumptions^12^ and of existing approaches.^8^ The development of MAPPIN'SDM involved the definition of process indicators of a consultation strategy maximizing patient involvement and meeting the criteria of evidence‐based patient information^19^ and the definition of detailed criteria for scoring more or less skilled performance of these indicators.^18^ Besides theory, this process was informed by insight into clinical practice via both an extensive pool of consultation recordings and intensive close co‐operation with doctors and patients at our unit.^21^ Basically, the doktormitSDM concept is a continuation of these discussions with the practitioners in which we shared our in‐depth insight into their communication using terms of the MAPPIN'SDM method. Together, we strove to identify the optimal strategies to fairly and efficiently involve the patient in making medical decisions. At its core, doktormitSDM is a practitioner‐trainer discourse on patient involvement, using an actual consultation recorded by the practitioner and its MAPPIN'SDM analysis by the trainer. The trainer provides measurement‐based supportive feedback in line with the concept's assumptions. Using simple didactic methods, the doctor is then stimulated to see the doctor–patient dialogue from a third person's perspective This is intended to enable doctors to evaluate, refine and develop their own communication behaviour, and thus to take long‐term control of their own learning.

#### 2.2.3 Didactic considerations

##### Making the training feasible

The impact of the programme would be limited by its compatibility with the participants’ daily routines. As regards practical issues, feasibility is challenged by the amount of time needed and the distance between the training site and the workplace. In addition, marginal discrepancies between the training content or context issues and the doctors’ reality might, from the doctors’ point of view, give reason to adopt a rejecting stance. For example, a doctor participating in a conference workshop might argue that an example provided in a roleplay is of no practical use because the consultation setting in his/her clinical reality is slightly different. Such dynamics might indicate strategies to reduce cognitive dissonance.^22^ The secret in preventing devaluation of training content caused by dissonance reduction lies in facilitating desirable strategies of dissonance reduction instead, in particular behaviour change. We presumed that behaviour change was most likely to happen when the interactive part of the training was provided at the participant's ward (in situ) and shaped and structured as an ultrashort unit of 15 minutes. Conflicts with daily duties were thus usually avoided. Rather than drawing the trainee's attention to external examples, the core component of the training used communication samples from the trainee's own daily work. The attitude of trainers towards trainees was open and non‐judgemental.

##### Overcoming barriers

During the modelling and piloting phase, we had learned about specific barriers clinicians typically face when intending to employ patient involvement behaviour. To achieve behaviour change, doktormitSDM has to address attitude‐related beliefs as well as misconceptions regarding subjective social norms and individual behaviour control.^23^, ^24^ For instance, participants might be open‐minded regarding the SDM approach but, as a result of their medical training, could expect to be quantitatively evaluated in the face‐to‐face feedback sessions. This misconception would mean a barrier to implementation of the target behaviour, as it would interfere with the autonomous role in the learning process intended for the trainee. This barrier adds to subjective social norm barriers arising from the way the information source, here the trainer and the training concept, is evaluated by the participants. For instance, a common view in the medical community is that physicians can just learn from physicians. Any feedback from a non‐medical communication expert might be at risk of being devalued. Learning, discovering and testing a new communication approach might also be inhibited by medical doctors’ anticipation of quantitative evaluation, which is caused by the socialization in traditional medical training. To open the doctors’ minds, the feedback must therefore be recognizable as both highly professional and precise in its detailed descriptions and explicitly qualitative. For example, the trainer might indicate detailed awareness of the consultation course, but instead of valuing certain actions as good or bad, he or she should ask questions to stimulate the trainee's self‐observation and self‐reflection.

Within the didactic of the doktormitSDM training, the way to handle the sensitive issue of control is considered the key to the participants’ learning potential. Having in mind that medical doctors are used to being in the driver's seat, the following hypotheses were guiding didactic decisions: involuntary loss of control, such as taking away familiar behaviour patterns, might impede behaviour change. Voluntary relinquishment of control, as evident in delivering one's own work samples (videos) to a communication analyst, implies huge potential for a fruitful learning process. Re‐transferal of control to the participants in terms of transparency regarding the methods used for analysis, sovereignty of judgement, encouragement, suggestions and discourse might strengthen the idea of partnership between trainer and trainee and implicitly provide a model of a good doctor–patient dialogue.

##### Achieving sustainable transfer into daily practice

**Figure 2:**
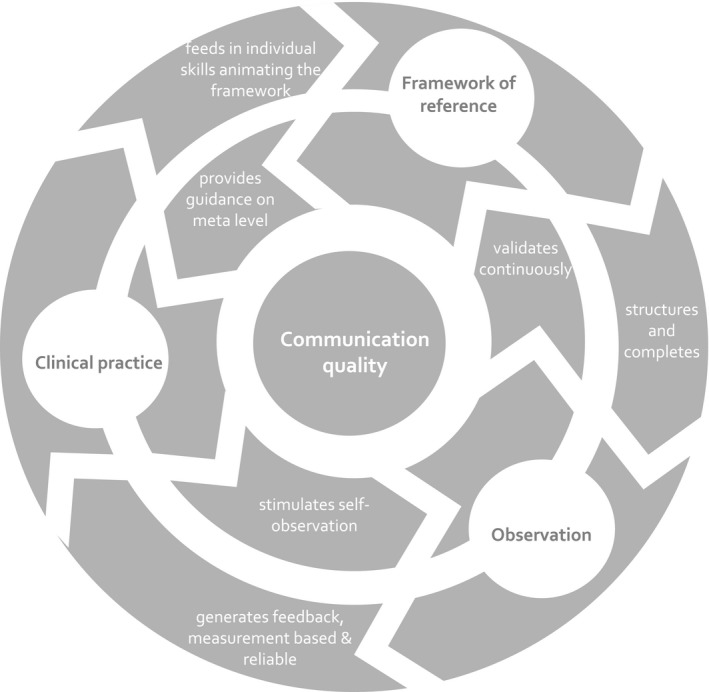
Didactic framework for developing communication quality. The diagram illustrates the interaction of the three key components in the doktormitSDM didactic strategy: the framework of reference, which is the MAPPIN’SDM taxonomy, the observation and the clinical practice

Development of a partnership is also the basis upon which strategies are employed to transform the initial learning within the minimal intervention into a process of continual development employing the same ideas and methods. These strategies are about sharing the innovative and evidence‐based approach. Particularly, by sharing the results of the video analysis, the trainer invites the trainee to take an analytical viewpoint and provides insight into how the structure of each consultation is approached; for example, trainees are given the opportunity to watch sequences of their own recordings in the third person role. Repeatedly, trainees are encouraged to express reflections on their own strategies and alternative solutions. The training thus aims to induce self‐observation using the given reference framework even in the absence of the trainer (Figure 2).

##### Tailoring the training individually

Although everybody agrees how important communication is, a closer look at this general attitude reveals a big variety of beliefs and motivation. Thus, it can be challenging to direct attention to the particular communication issue and to excite the participant's curiosity. The doktormitSDM training therefore puts emphasis on individualization. Although using the MAPPIN'SDM taxonomy as a background structure, the programme consistently applies an intra‐individual reference norm. Rather than: “How is your performance compared to others?” the leading question is: “What would your way to do this look like?” Trainers have the competence to adapt to each trainee's SDM level and particularly try to improve pre‐existing skills. Individual barriers are explored and discussed. Moreover, trainers continuously evaluate the trainees’ capacity and tolerance so that feedback can be stopped before resistance rises.

Summarizing, the didactic proceedings emphasize use of the communication (measurement) framework for applying observation to clinical practice. Participants are encouraged to recognize and discover their own skills to incorporate the framework of reference. In this regard, doktormitSDM is a learning concept, as new observation always leads to refinement of the framework. The relations reflecting the underpinning didactic concept are shown in Figure 2.

### 2.3 Sample

The pretest used a convenient sample of 10 doctors and a consultation sample of 40 doctor–patient consultations. The sample of doctors was composed of specialists at several outpatient clinics of the Hamburg University Medical Center and medical practices in Hamburg, Germany. The behaviour sample comprised forty recordings of consultations. Consultations were eligible if they included a medical decision on either treatment or diagnostics that involved choosing between more than one option, which could also be the option of doing nothing or postponing an action. Patients were eligible regardless of any additional criterion. Recordings were to be made in accordance with the individual schedule provided, in particular after each single intervention component. Within a particular slot in the schedule, the doctors were asked to record the next eligible consultation. The study team maintained close contact with the participants and provided reminders by telephone. Selection of consultations for recording could, however, be affected by other factors not under the control of the study team, such as refusal to give informed consent by the patient, or memory or stress on the doctors’ side. Recordings already made could not be deleted afterwards.

### 2.4 Measurement

For sample description purposes, specialty, gender, work experience and level of previous SDM training of the participating doctors, gender of the patient as well as medical domain, length and medical decision topics of the consultations were documented. Doctors gave informed consent at the beginning of the study and thereby agreed to the use of four subsequent consultation recordings. Agreements with the participating doctors also covered recruiting of patients, obtaining their informed consent and instructing them with regard to the use of the questionnaire.

To study feasibility and subjective usefulness, the participating doctors were asked to fill in an additional one‐page questionnaire after finishing the training. Using 27 items, the questionnaire aimed particularly to explore the extent of the participants’ use of the materials provided (two items), comprehensibility of the materials (three items), attitude and change of attitude towards patient involvement (two items), usefulness of the training in general and of each single component including the questionnaire as a didactic component (seven items), self‐assessment of patient involvement (four items), need for a change of emphasis amongst (four items) or order of the three components (four items) and feedback to the intervention team (one item). Three items used free text formats, and 12 items used a four‐point Likert scale. Emphasis on the components could be rated as “too much,” “not enough” or “perfect,” and suggestions for ordering could be provided by indicating one of three positions. Self‐assessment of performance was rated on a scale from 0 “SDM not present” to 6 =“excellent” (Table 1).

**Table 1:** Questionnaire to evaluate the feasibility of the doktormitSDM training module

Questionnaire for evaluation of the feasibility of the doktormitSDM training module				
Topics addressed		Range	Mean	SD
Extent of use	Manual	0=“not at all” to 3=“intensive”	2.1	0.3
	Video tutorial	0=“not at all” to 3=“intensive”	2.0	0.5
Comprehensibility	Questionnaire	0=“not at all” to 3 =“fully”	2.4	0.7
	Manual	0=“not at all” to 3=“fully”	2.6	0.5
	Video tutorial	0=“not at all” to 3=“fully”	2.7	0.7
Attitude: SDM desirable	General	0=“not at all” to 3=“fully”	2.8	0.4
	Change	0=“not at all” to 3=“fully”	1.2	0.1
Usefulness of the training	General	0=“not at all” to 3=“fully”	2.4	0.7
	Manual	0=“not at all” to 3=“fully”	1.9	0.8
	Video tutorial	0=“not at all” to 3=“fully”	2.2	0.8
	Feedback	0=“not at all” to 3=“fully”	2.7	0.7
	Questionnaire	0=“not at all” to 3=“fully”	2.4	0.5
Self‐assessment of SDM performance	Consultation 1	0=“not present” to 6=“excellent”	2.8	0.7
	Consultation 2	0=“not present” to 6=“excellent”	2.6	0.5
	Consultation 3	0=“not present” to 6=“excellent”	2.2	0.4
	Consultation 4	0=“not present” to 6=“excellent”	2.1	0.2
Extent and emphasis of module and components	Manual	Too much/not enough/perfect	5=too much manual	
	Video tutorial	Too much/not enough/perfect	4=not enough video tutorial	
	Feedback	Too much/not enough/perfect	5=not enough feedback	
Change order of components	Manual	Second, third	Summarizing comments regarding order of the components: “present the manual last and begin with the feedback”	
	Video tutorial	First, third		
	Feedback	First, second		

The topics used and the results gained through the doctor questionnaire completed by the finishers of the training.

The extent of patient involvement was assessed from three measurement perspectives using five scales of the original German version of the MAPPIN'SDM inventory.^18^ MAPPIN'SDM includes three observation scales for doctor, patient and the doctor–patient–dyad (which is the unit made up of doctor and patient). Additionally, the inventory includes two corresponding questionnaires for doctor and patient. All five scales address an identical set of 15 indicators of patient involvement^18^ (Table 1). Each indicator is represented by one item, which is scored from “0” (“the indicator is not present”) to “4” (“the indicator is present at an excellent standard”).

The unit of analysis to assess patient involvement was the decision sequence within the medical consultation. To make sure that the measures of patient involvement were applied to the same unit, doctor and patient had to agree upon one decision as the index decision they would refer to when completing the questionnaire. To prepare the observer ratings, sequences including the index decision were coded a priori with regard to timeline and the set of available options. If necessary, medical expertise was requested to affirm the set of available options. Sequences were selected in random order during the rating procedure.

The two raters coding the communication material in this study were successful finishers of a previous coder training.^25^ Ratings were assisted by a comprehensive manual providing detailed examples for the scoring of each item. Both raters coded the material independently to allow for calculation of inter‐rater agreement. In a second step, a consensus rating was agreed upon by discourse, which was used in the analyses in this study. By use of a pseudonym and randomized selection for coding, raters were blinded towards questionnaire data and the doctors’ level of SDM training. MAPPIN'SDM questionnaires were completed by doctors and patients immediately after the consultations. To become familiar with the questions they had to answer afterwards, doctors using the questionnaire for the first time and patients were asked to read through it prior to the consultation.

### 2.5 Analyses

Descriptive data characterizing the study sample were collected. SDM was assessed in 11 separate series: three for each rater using the MAPPIN'SDM observer scales (MAPPIN‐O_doctor_, MAPPIN‐O_patient_ and MAPPIN‐O_dyad_), a consensus judgement for each of the three MAPPIN'SDM observer scales and two series from the questionnaires (MAPPIN‐Q_doctor_, MAPPIN‐Q_patient_). Data series from each observer were used to calculate pairwise inter‐rater reliabilities using Spearman's ρ correlation coefficients for each item and for the scale as whole.

Based on the consensus values on the MAPPIN'SDM observer scales and the two questionnaire scales, mean scores were calculated for each training level (C1 to C4). Using Wilcoxon signed‐rank tests for nonparametric distributions, improvement from C1 to C4 was tested for statistical significance (α=0.05, one tailed). In addition, Spearman's ρ correlation coefficients were calculated between duration of decision sequences and extent of patient involvement.

The doctors’ feedback provided in the feasibility questionnaire was aggregated item‐wise using mean values and standard deviations where appropriate.

The relation between the time used for making decisions and the quality of the communication was assessed by calculating Pearson's correlation coefficients between the length of the pure decision sequences within the consultations and the mean scores for patient involvement drawn from the MAPPIN'SDM scales.

All analyses were conducted using SPSS version 17.

## 3 RESULTS

### 3.1 Descriptive results

Amongst the 40 patients participating in the study, 22 were male. The 10 doctors (seven of whom were male) were specialists in neurology (4), dental (3) and internal and general medicine (3). A big variety of medical problems and treatment was discussed as well as the appropriate diagnostic medical options. This ranged from consideration of immunotherapy treatment or the procedure to confirm a diagnosis of multiple sclerosis to problems related to tooth loss, treatment of serious cardiovascular disease and questions related to prophylactic interventions such as vaccinations. The length of consultations ranged from 2.5 to 51 minutes (mean 20.5 minutes), while the length of decision sequences ranged from 2.5 to 38.8 minutes (mean 14.8 minutes).

### 3.2 Feedback by doctors

According to subjective reports given by the participants after finishing the training, doctors made use of the materials provided in addition to the feedback sessions. Although the participants stated that the training did not significantly change their attitude towards SDM (which was already very positive for most participants), the intervention in general and each component were perceived as comprehensible and useful. Following both Likert scale ratings and the comments provided as free text, the face‐to‐face feedback was considered most supportive. Contrasting the observer ratings and the results of the doctors’ own detailed assessment in the questionnaire, self‐assessment indicated a decrease in communication quality from the first to the last consultation. Recommendations for improvement of the programme quite consistently suggested a more prominent role for face‐to‐face feedback with regard to both order and emphasis of the components (Table 1).

### 3.3 Quality of measurement

Inter‐rater reliabilities were high to excellent in the observer scales (MAPPIN‐O_doctor_ *r*=.87, MAPPIN‐O_patient_: *r*=.81, MAPPIN‐O_dyad_: *r*=.74). Internal consistencies of the two questionnaire scales were high (Cronbach's alpha: MAPPIN‐Q_doctor_=.94, MAPPIN‐Q_patient_=.94).

### 3.4 Quality of the communication

**Figure 3:**
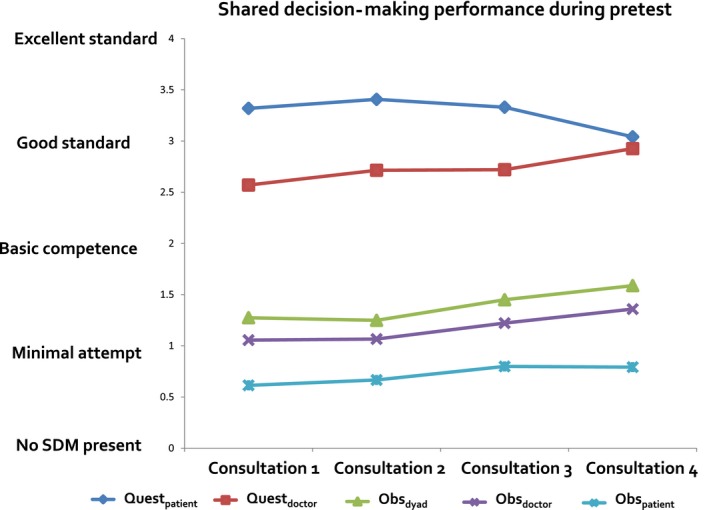
Shared decision‐making performance over the training period. The diagram shows level and development of communication quality from baseline to end of training. The lines represent the three MAPPIN’SDM observer scales and the two questionnaires

According to independent observers, before and after training the consultations showed poor patient involvement (mean MAPPIN‐O_doctor_=1.2, SD=0.4, MAPPIN‐O_patient_=0.7, SD=0.3, MAPPIN‐O_dyad_=1.4, SD=0.4; range 0 to 4), while the same communication was evaluated better by the parties directly involved (mean MAPPIN‐Q_doctor_=2.7, SD=0.7; MAPPIN‐Q_patient_=3.3, SD=0.6). The communication improved during training from the first to the fourth consultation according to observers rating the performance of doctors (MAPPIN‐O_doctor_: *P*=.056) and doctor–patient–dyads (MAPPIN‐O_dyad_: *P*=.065) and to doctor questionnaires rating the dyads’ performance (MAPPIN‐Q_doctor_: *P*=.023). No improvement was observed in the patients’ active involvement (MAPPIN‐O_patient_: *P*=.109); in addition, the patients’ evaluation did not indicate improvement of the communication during SDM training (MAPPIN‐Q_patient_: *P*=.145). Mean scores on all MAPPIN'SDM scales except for the two patient scales ascended continuously over the four measurement points (Figure 3).

### 3.5 Length of consultations

In this study, length of decision talk was associated with quality of the communication as assessed by SDM observers and the doctors themselves (MAPPIN‐O_doctor_: *r*=.69, *P*<.001, MAPPIN‐O_patient_: *r*=.37, *P*=.03, MAPPIN‐O_dyad_: *r*=.64, *P*<.001, MAPPIN‐Q_doctor_: *r*=.33, *P*=.052, MAPPIN‐Q_patient_: *r*=.33, *P*=.052), while patients’ assessment of the communication was not related to the length of the decision talk sequence (MAPPIN‐Q_patient_: *r*=.07, *P*=.700).

## 4 DISCUSSION

The study introduced a short intensive training module for doctors to enhance communication in terms of patient involvement in making medical decisions. The training was shaped practically and designed didactically to facilitate a sustainable learning process. Typical barriers to this process are considered in detail.

To participate in the doktormitSDM training, doctors do not need to leave their workplace and only have to invest about 2 hours of study time and 15 minutes to receive the face‐to‐face feedback. The training was considered comprehensible and useful. No incompatibilities with on‐going clinical work were observed or reported. Even this minimal application of the doktormitSDM approach caused significant enhancement of the communication quality.

It is debatable whether increased demonstration of behaviours considered essential in the MAPPIN'SDM taxonomy means a substantial improvement of the communication. In absolute terms, use of the particular skills increased on average by only one thirteenth of the entire range of the SDM scale. Patient activity indicating SDM was not observed to a greater extent after the training. And, interestingly, in their global rating provided in the feedback questionnaire, the doctors retrospectively judged their own consultations more and more critically with regard to training progress. Data on intercorrelation of the scales within the MAPPIN'SDM inventory are presented in another paper.^18^ The patients’ judgements, which are presumably less biased owing to the fact that all patients only participated once in the study, do not reflect an improvement in communication. Although the patients’ tendency to give lower ratings with increasing training level might, considering the small sample size, be due to a random variation, it is hard to ignore (Figure 3). The discrepancy between the gain in skills and the evaluation of the patients might reflect the incomplete implementation of new behaviours in the consultations. We have not measured whether adaptation of new behaviours was accompanied by loss of other behavioural habits such as providing emotional support or the impression of certainty. The drop in patients’ ratings with increased training could reflect a general reduction in the perceived empathy of the doctor. However, as the doktormitSDM training is designed to stimulate continuous self‐observation, it is also possible that the study effects consolidate further on. The relation between communication quality and consultation length is an important parameter frequently used in discussions about implementation of SDM. Most of the existing evidence in this regard has been gained from studies employing decision aids in medical consultations. In the current review of efficacy of patient decision aids, results related to length of consultations are ambiguous but predominantly indicate patient involvement to require additional time.^2^ Little is known about consultation time requirements in the longer run. However, we consider the positive correlation found in our study to be far from conclusive. Firstly, with regard to the big variation of consultation length within the sample and between the medical domains, and secondly due to the relatively small absolute improvements, we assume that these correlations are most likely to be caused by confounders, such as motivation and social desirability issues, rather than the communication approach.

In view of the small scale of the intervention, the study is minimalistic with regard to sample size. A follow‐up power calculation based on actual distribution parameters showed that the empirical differences would have become universally significant with a doctor sample twice the size. Due to internal dependency of the data and the absence of a control group, efficacy would have been hard to interpret. This also applies due to a potential selection bias caused by convenient sample recruitment regarding both the participating doctors and the choice of appropriate behaviour examples for recording.

As recent reviews^5^, ^17^, ^26^ indicate, training and teaching methods in shared decision making are still underdeveloped. This situation is contrasted by high activity in developing patient decision aids.^2^ This imbalance might imply that developing media‐based tools is considered easier than addressing communication itself. Another recent review seems to show that shared decision making will not be adequately implemented until strategies approaching from various directions, such as from the patients’ and the health professionals’ side, are used to complement each other.^6^

Meanwhile, another training curriculum for SDM has been evaluated with German doctors.^27^ The curriculum turned out to be effective in improving skills classified with the OPTION scale, which partly covers essential SDM indicators.^28^ Results were, however, gained within a highly selected and reduced sample remaining at follow‐up. The dramatic loss to follow‐up reflects the poor feasibility of the intervention. In contrast to the authors’ argumentation that the dosage of 12 SDM teaching sessions might have been too little, we consider that this involves too much effort to have a realistic chance of implementation. In this regard, the doktormitSDM training seems promising as a method for training specialist doctors in the German health system. Although developed together with medical doctors, it is applicable without limitation to other health professionals such as nurses or physical therapists. Moreover, the doktormitSDM approach is adaptable to various settings, media and time frames. The one‐on‐one didactic method has recently been translated into an online format.^29^, ^30^ Currently, much effort is being made to develop communication curricula for medical students.^31^, ^32^ However, the didactic strategies being used are not necessarily transferable to professional training. In a current trial, an adapted online tutorial application of the doktormitSDM curriculum is therefore being tested with medical students.

Training programmes on SDM require thorough consideration of current clinical communication routines. The “one doctor – one patient” setting is no longer the most common setting for making key decisions in many medical domains and has been replaced by virtual or real multidisciplinary settings such as tumour boards.^1^ Didactic strategies of training have to respond to these new challenges.

## 5 CONCLUSION

The new SDM training module has proven feasible and was considered important and supportive by the participating doctors. Used as a minimal intervention, this training approach has shown potential to positively affect the communication quality in terms of patient involvement. These results suggest that further evaluation will be worthwhile. The conclusions of this study have led to a revised version of the doktormitSDM training which is now being tested within a randomized controlled trial.

## CONFLICT OF INTEREST

We have no conflict of interests to declare.
